# Silage Characteristics of Selected Forage Maize Varieties Harvested in Sole and Forage Legume Mixtures

**DOI:** 10.1155/tswj/2270637

**Published:** 2025-03-28

**Authors:** Poloko Mosebi, Moeketsi Ntakatsane, Tumelo Nkheloane, Tumelo Manyeli, Palo Loke

**Affiliations:** ^1^Department of Animal Science, Faculty of Agriculture, National University of Lesotho, Maseru, Lesotho; ^2^Department of Extension, Food Security and Nutrition, Ministry of Agriculture, Maseru, Lesotho; ^3^Department of Soil Science, Lesotho Agricultural College, Maseru, Lesotho

**Keywords:** fermentation products, forage silage, microbial count, nutrient composition

## Abstract

The present study was conducted to evaluate the ensiling characteristics of selected forage maize varieties harvested in sole and forage legume mixtures. Two different forage maize varieties were harvested in different forage systems, that is, sole maize, maize + common vetch and maize + lablab intercrops, and ensiled in small-scale silos. After ensiling, samples were collected to examine the nutrient composition, fermentation quality and microbial population. Ensiled forage varieties harvested in sole forage systems had a significantly (*p* < 0.05) lower dry matter and crude protein contents (31.18% and 7.15% DM, respectively) than that from forage legume mixtures. Forage legume mixtures had significantly (*p* < 0.05) lower neutral detergent and acid detergent fibre contents (24.42% and 35.53% DM, respectively) and higher water-soluble carbohydrates (12.34% DM) in silage of selected varieties than sole forage systems. The pH value and ammonia nitrogen content of ensiled forage varieties were lower, while lactic acid production (7.25% DM) was greater in forage legume mixtures than in sole forage system. Lower acetic and propionic acid and higher butyric acid contents (1.06%, 0.84% and 0.46% DM, respectively) were observed in sole forage system for silage of selected varieties compared to forage legume mixtures. Sole forage systems showed lower numbers of lactic acid bacteria and higher populations of enterobacteria (4.31 and 4.34 log_10_ cfug^−1^, respectively) in silages of selected varieties than forage legume mixtures. The study concluded that ensiling forage materials harvested in forage intercrops have a positive effect on the silage quality. Therefore, the recommended forage system for ensiling is forage legume mixtures.

## 1. Introduction

Maize is one of the most important crops in smallholder farming, as it is the main source of food for people and is used for animal feed. It is the most important cereal grain for small-scale farmers, providing nutrients for humans and animals and serving as a basic raw material for the production of starch [1]. As a food, the whole grain may be processed by dry milling techniques to give a relatively large number of intermediary products, such as maize meal and maize flour [2]. In recent years, even in smallholder farming in which maize is a staple food, more of it has been used as an animal feed ingredient and compound feed for poultry, pigs and ruminant animals [3]. The by-products of maize from dry milling such as seed coat or pericarp are mainly used as animal feedstuff and by-products such as maize gluten are also used as a feed ingredient [4].

Maize has different varieties that are more important for small-scale farmers because of their multiple uses. Other maize cultivars are used for animal feedstuff, whether as grain or as the whole plant, which can either be baled or made into a more palatable silage [5]. After harvesting the grain, the dried leaves of maize are still used today to provide relatively good forage for ruminant animals owned by many small farmers [6]. The green maize plant, made into silage, has been used with much success in the dairy and beef industries [7]. Worku et al. [8] also reported that silage maize is the basic feed in cattle fodder rations for producing milk and meat.

Although maize plant residues are a valuable source of fodder for livestock, conserving green maize forage has been recognised as one of the major challenges, especially in smallholder farming systems. Small-scale farmers are subject to some drawbacks, and limited knowledge to assist in the silage-making process is the most crucial factor. During ploughing of crop fields, the main focus in intercropping maize with legumes has been to increase grain yields of crops, but little attention has, until recently, been paid to the benefits of intercropping maize with forage legumes for forage conservation methods, most importantly silage making. Smallholder farmers are very familiar with the use of crop by-products as animal feed but less familiar with the use of intercropping maize with forage legumes in the warm season (summer months) for the production of high-quality silage designed for livestock feeding in the dry season.

Fresh forage crops from intercropping systems such as maize can be preserved by ensiling. The technology of ensiling forage crops has been strongly regarded as a means of preserving large quantities of biomass feedstocks for feeding animals after cropping seasons [9]. This method has the potential to maintain the nutritional status of fodder during the dry season when green fodder is not available and ensiled forages are highly valued as animal feed. Similarly, there has been growing interest in the production of forages in recent years because they have the potential to be used in silage-making processes. Intercropping systems are considered the most common cropping method used to provide forage with more than one species in the same field for silage making [10].

In this study, different forage maize varieties harvested from sole forage legume intercrops through the silage-making technique are integrated to provide practical solutions to enhance livestock feed quality given that a sustainable supply of quality forage is a concern in dry seasons. The information generated provided the findings that can be used by smallholder farmers to adopt or in some cases improve their forage production practices, harvesting and post-harvesting handling techniques. The findings of this work also generated information vital for contribution of a new knowledge to the scientific community. The work also contributed the information that is a valuable resource for policy makers, researchers and practitioners in agricultural and animal sciences. In this study, it was hypothesised that forage maize varieties do not necessarily have different chemical composition and silage characteristics when ensiled from sole and forage legume intercrops. The present study was conducted to evaluate the effect of sole and forage legume intercrops on nutritive value, fermentation quality and microbial composition of ensiled forage maize varieties.

## 2. Materials and Methods

### 2.1. Study Site

A field experiment was conducted at St. Michael (29° 25′ 38S, 27° 40′ 50E, and altitude 1700 m above sea level) for two growing seasons (2022-2023) under rainfed conditions. The site lies in a lowland region of Lesotho characterised by cold winters and warm summers. Winters are generally characterised by warm to moderate temperatures during the day, with a sudden cold temperature just after sunset. The average annual temperature ranges between 15.2°C and 25.6°C. Rainfall ranges from 500 to 1000 mm per annum, with the highest values in the summer season [11]. The predominant soils of the area are classified as fersiallitic soils with an orthic A horizon [12]. Common vegetation included grass species and forbs.

### 2.2. Agronomic Practices

Two maize (*Zea mays* L.) varieties were grown as a sole crop and mixtures with common vetch (*Vicia sativa* L.) and lablab (*Lablab purpureus*) in 10 × 5 m plots and replicated three times. Maize was considered as the main crop while common vetch and lablab as an intercrop companion crop. Plant varieties used in the experiment were Rongai for lablab, Morava for common vetch and Pannar 12 and DKC 7372 for maize. A basal fertiliser (6:3:4 NPK) was applied to all maize plots at a rate of 75 kg ha^−1^ at sowing. Legume-only plots received a combination of P and K at 80 kg ha^−1^. The spacing for sole maize (SM) was 75 cm × 30 cm between rows of plants, respectively, and that for sole common vetch and lablab was 40 cm × 10 cm between rows of plants, respectively. Lablab and common vetch were intercropped between two maize rows at 37.5 cm away from the maize row with interrow and 10 cm intrarow spacing. The plant density of maize was 40,000 plants/ha, and the forage legumes (lablab and common vetch) were planted at 70,000 plants/ha. All necessary agronomic practices (weed, pest and disease control) were kept uniform for all experimental plots during the cropping season. Selected maize varieties samples were harvested at 80 days after sowing, and the chemical composition of forage samples was assessed directly prior to ensiling. The characteristics of the sole and intercrops forage materials are described in Table 1.

### 2.3. Silage Preparation

After harvesting, Pannar 12 and DKC 7372 maize variety samples were separately ensiled. Silage was prepared from each variety harvested from SM and in mixtures of maize + common vetch (MC) and maize + lablab (MB) from the intercrops. The forage material was ensiled in small-scale silos, 50 L polyethylene drums. The harvested forage materials were chopped to lengths of approximately 3–4 cm using a forage cutter and wilted overnight to reduce moisture content to 50%–45%. The forage materials were ensiled in the small-scale silos to a capacity of 20.0 ± 0.5 kg. Once filled with forage material, the small-scale silos were compacted using a pressing apparatus to expel air to ensure sustainable anaerobic conditions. The experiment was designed as a completely randomised design with a 3 (forage systems) × 2 (selected forage maize varieties) factorial treatment arrangement and three replicates. The small-scale silos were then incubated at room temperature (±25°C–30°C) for 60 days.

### 2.4. Silage Fermentation Characteristics

The silages were evaluated after 60 days of ensiling. Samples were collected and freeze dried using an Alpha 1-4 LSC freeze drier (Martin Christ Freeze Dryer, Germany) at −60°C and at a pressure of 0.011 mbar for 24 h. Dried samples were ground in a laboratory mill (Thomas Wiley model 4, Arthur Thomas & Co.) with a 1 mm screen and stored for chemical analysis. Dry matter (DM) and crude protein (CP) were determined using AOAC [13] procedures; neutral detergent fibre (NDF) and acid detergent fibre (ADF) contents were determined following the Van Soest, Robertson and Lewis [14] method. Water-soluble carbohydrate (WSC) content was determined using McDonald, Henderson and Heron [15] procedures.

The determination of fermentation quality and microbial counts was conducted on the day of silo opening. Immediately after opening the small-scale silos, 40 g of each fresh silage sample was weighed and placed into a sealable container with 350 mL of distilled water. The container was closed and shaken for 6 h at 180 rpm using a horizontal shaker. The silage extract was then filtered through four layers of cheesecloth directly after sample preparation. An electrode pH metre in a water-based solution was used to measure pH. Silage extracts (25 mL) were placed in a stomacher blender for 3 min and filtered through Whatman filter paper to determine silage ammonia nitrogen (NH_3_-N) [16]. For lactic acid and volatile fatty acid (VFA) analysis, silage extracts (2 mL) were filtered and acidified with metaphosphoric acid (25%) to reduce the pH of the extracts. Samples were then centrifuged for 15 min using a capillary column over a temperature of 40°C to determine lactic acid and VFA concentrations, including acetic acid, butyric acid and propionic acid [17].

The plate culture method was used to determine microbial counts of the fresh silages. Twenty grams of each sample was weighed and shaken well with 100 mL of sterilised distilled water and diluted in a sodium chloride solution (0.90%). Enterobacteria were determined on a plate of violet red bile agar with lactose after anaerobic incubation at 35°C for 24 h. Lactic acid bacteria (LAB) were counted on an agar plate of Lactobacilli MRS broth after incubation in an anaerobic box at 40°C for at least 24 h. For analysis of the microbial population, colonies were counted, and their numbers were expressed as viable numbers of microorganisms in colony forming units per gram of fresh matter (cfug^−1^) and transformed into log_10_ of cfug^−1^.

### 2.5. Statistical Analysis

Analyses of variance were performed on the data using the general linear model (GLM) procedures of the Statistical Analysis System [18]. The variance was apportioned to the forage system, maize variety and their interaction as expressed in the model. The dependent variables were chemical composition, fermentation quality and microbial populations. Treatment means were compared using a protected LSD (*p* < 0.05) test.

The model is presented as follows:

(1) $$\begin{array}{c} Y_{ijk}=\mu +\alpha_{i}+\beta_{j}+{\alpha \beta }_{ij}+\varepsilon_{ijk}, \end{array}$$

where *Y*_*ijk*_ is the variable response; *μ* the general mean; *α*_*i*_ the forage system effect; *β*_*j*_ the maize variety effect; *αβ*_*ij*_ the forage system by maize variety interaction; and *ε*_*ijk*_ the residual error.

## 3. Results

### 3.1. Forage and Silage Chemical Composition

The chemical composition of sole and intercrop forage materials before ensiling is presented in Table 1. The DM for the forages ranged from 32.78% to 38.74%, and the sole forages had lower values than mixtures (*p* < 0.05). The CP content varied between 7.15% and 8.58% DM and the mixtures had higher levels than the sole forages (*p* < 0.05). The NDF and ADF contents ranged from 24.22% to 34.54% and 35.95% to 38.26% DM, respectively; the highest and lowest values (*p* < 0.05) were observed in sole and mixtures, respectively. Across selected forage varieties, variety 1 (Pannar 12) had the highest (*p* < 0.05) DM of 36.05%, followed by variety 2 (DKC 7372) with a value of 34.15%. The CP content ranged between 6.95% and 7.77% DM and the lowest value was found in variety 2. The NDF concentration varied between 25.42% and 28.55% DM and the highest values (*p* < 0.05) were observed in variety 2. Variety 2 had the lowest ADF content of 37.89% DM compared to variety 1 (38.02% DM). The forage systems × selected varieties interaction for chemical composition was statistically significant (*p* < 0.05). The sole forages had a significantly lower (*p* < 0.05) chemical composition in selected varieties than the mixtures.

The chemical composition of the silages is presented in Table 2. For the forage systems, the DM and CP contents ranged from 31.18% to 37.85% and 6.99%–8.15% DM, respectively. SM silage had lower (*p* < 0.05) DM content, followed by MC silage and the higher value (*p* < 0.05) was recorded in ML silage. MC silage had higher CP concentrations than ML and SM silages. The DM and CP contents in the forage varieties were 32.61%–34.71% and 6.36%–7.18% DM, respectively. Variety 1 had higher (*p* < 0.05) DM content than variety 2, and the CP content also showed a similar trend. Different forage systems showed significant effects on DM and CP contents for the silage of selected varieties. The ensiled forage varieties in sole forage system (SM) had a significantly lower (*p* < 0.05) DM and CP contents than the silages in forage legume mixtures (MC and ML).

The NDF, ADF and WSC ranged between 23.12%–33.25%, 35.28%–37.08% and 11.22%–12.71% DM across forage systems, respectively. MC and ML silages had lower (*p* < 0.05) NDF and ADF values than SM silages. WSC concentration of the SM silage was less (*p* < 0.05) than that of MC and ML silages. Across forage varieties, the NDF, ADF and WSC values varied from 24.91% to 27.82%, 37.01% to 37.21% and 11.35% to 12.46% DM, respectively. Variety 1 had lower (*p* < 0.05) NDF content and higher ADF and WSC values than variety 2. Different forage systems and varieties showed significant effects (*p* < 0.05) on NDF, ADF and WSC concentrations. The forage legume mixtures had significantly lower (*p* < 0.05) NDF and ADF contents and higher (*p* < 0.05) WSC production in silage of selected varieties than the sole forage system.

### 3.2. Silage Fermentation Products

The fermentation profile of the silages is presented in Table 3. pH, NH3-N and lactic acid across different forage systems ranged from 3.08 to 3.42%, 2.96 to 3.73% TN and 5.94 to 7.84% DM, respectively. The lowest (*p* < 0.05) pH values were found in ML followed by MC and SM forage systems. Lower (*p* < 0.05) NH_3_-N content was observed in the MC compared to other forage systems. The lactic acid production of SM was lower (*p* < 0.05) than that of MC and ML forage systems. pH, NH3-N and lactic acid production across forage varieties varied between 3.21 to 3.32%, 3.78 to 4.26% TN and 7.18 to 7.36% DM, respectively. Ensiled variety 2 had high (*p* < 0.05) pH values and NH_3_-N contents and low lactic acid concentrations compared to variety 1 silage. Different forage systems and selected varieties showed significant effects (*p* < 0.05) on pH, NH_3_-N and lactic acid production. The forage legume mixtures had significantly lower (*p* < 0.05) pH and NH_3_-N contents and high (*p* < 0.05) lactic acid production in ensiled varieties than the sole forage system.

The acetic, propionic and butyric acid production for different forage systems was 1.06%–1.88%, 0.84%–1.18% and 0.24%–0.46% DM, respectively. Acetic acid concentration was highest (*p* < 0.05) in the ML, followed by MC and SM forage systems. The highest (*p* < 0.05) propionic acid concentration was observed in MC compared to the other forage systems. The concentration of butyric acid was lowest (*p* < 0.05) in the forage legume mixtures than the sole forage system. The acetic, propionic and butyric acid concentrations across selected varieties ranged from 1.39% to 1.81%, 0.92% to 1.02% and 0.28% to 0.35% of DM, respectively. The ensiled variety 1 showed a higher (*p* < 0.05) acetic and propionic acid concentrations than the variety 2 silages. Lower butyric acid concentration was found in ensiled variety 1 compared to variety 2 silage. There were significant differences between the forage systems and selected varieties in terms of acetic, propionic and butyric acid concentrations. Lower (*p* < 0.05) acetic and propionic acid and higher (*p* < 0.05) butyric acid contents were observed in sole forage system for silage of selected varieties compared to forage legume mixtures.

### 3.3. Silage Microbial Counts

**Figure 1:**
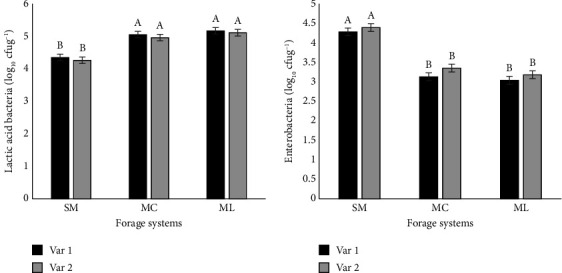
Influence of different forage systems on the microbial composition of forage maize silages. Error bar represents standard error of mean (SEM). Note: ^a-b^Values with different letters within the bar charts are significantly different (*p* < 0.05). SM, sole maize; MC, maize + common vetch; ML, maize + lablab; var 1, Pannar 12; var 2, DKC 7372.

The count of microbial colonies in the silages is presented in Figure 1. The LAB and enterobacteria across forage systems ranged from 4.31 to 5.14 and 3.11 to 4.34 log_10_ cfug^−1^, respectively. ML and MC forage systems showed higher (*p* < 0.05) growth of LAB compared to SM forage system. The highest (*p* < 0.05) enterobacteria count was observed in SM forage system followed by MC and ML forage systems. The LAB and enterobacteria across selected varieties ranged between 4.78 and 4.86 and 3.48 and 3.64 log_10_ cfug^−1^, respectively. The ensiled variety 1 produced higher LAB count and lower enterobacteria count compared to the variety 2 silages. The interaction of forage systems and selected varieties had significant effects on the population of LAB and enterobacteria. Sole forage systems showed lower (*p* < 0.05) numbers of LAB and higher (*p* < 0.05) populations of enterobacteria in silages of selected varieties than forage legume mixtures.

## 4. Discussion

### 4.1. Chemical Composition

Smallholder farmers use forage crops to feed their animals. However, forage materials are not widely ensiled in the form of silage making. Ensiled forage crop varieties produced from the forage cropping system can have the potential as a source of feed for livestock during the dry season. The use of different forage systems might result in distinct ensiling characteristics of selected forage varieties. Therefore, it is important to identify the best forage system and select suitable forage varieties to use for silage making. It is well known that DM and CP contents are important nutrient compositions reflecting the quality characteristics of silage [19]. In this study, sole forage silages had lower DM and CP content compared with silage mixtures. It is plausible that the quality of the ensiled forage from forage legume mixtures had a better chemical composition than the sole forage. Lai et al. [20] also reported higher DM and CP content in silage mixtures compared to other sole silages. Variety 1 silages had higher DM and CP concentrations than that of variety 2 silages. Kitaw et al. [21] also noted differences in the nutrient composition of selected ensiled maize forage varieties.

Generally, NDF, ADF and WSC can be influenced by the forage systems and activity of the fermentation during the ensiling process [22]. In the present study, lower NDF and ADF and greater WSC concentrations were produced in the silage mixtures compared to the sole forage silages. The reason might be the favourable conditions for the fermentation process and improved nutrient composition of ensiled forage materials. This agrees with Cheng et al. [23] who reported high WSC levels and low fibre components in ensiled soybean–maize mixtures. In the current study, the WSC value of ensiled forage variety 2 was lower while the NDF content was higher compared to forage variety 1 silage. A possible reason could be explained by lower DM and CP content observed before the ensiling process in variety 2. It has been reported that fermented forages have more WSCs and lower fibre content due to their abundant DM and CP concentrations before the fermentation process [24].

### 4.2. Fermentation Profile

Quality characteristics of ensiled forage and improved fermentation process are indicated by lower pH, decreased NH_3_-N and greater lactic acid concentration in silage [25]. In the present study, the silage mixtures had the lowest pH and low NH_3_-N content, but the highest lactic acid compared with sole forage silage. High production of LAB and lower enterobacteria produced in silage mixtures might have facilitated the release of lactic acid and lowered the pH values and NH_3_-N content. These results are similar to those reported by Li et al. [26] for silage mixtures treated with different additives and LAB. Moreover, pH tended to be higher in the ensiled forage variety 2 than in the forage variety 1 silage, NH_3_-N production was lower in the forage variety 1 than in the forage variety 2 silages and lactic acid production was greater in the forage variety 1 than in forage variety 2 silages. Similarly, Yang et al. [27] reported better fermentation quality as evidenced by higher lactic acid and lower pH and NH_3_-N concentrations when ensiling a mixture of maize and soybean.

Preservation of ensiled forage crops is supported by low pH values and organic acids such as acetic acid, propionic acid and butyric acid. Low pH and organic acids possess antifungal activity which improves fermentation and reduces the spoilage of microorganisms during the ensiling process [28]. In the current study, the concentration of acetic and propionic acid was lower and that of butyric acid was higher in sole forage silages than in silage mixtures. A possible reason for a lower concentration of acetic and propionic acid levels in sole forage silages could be less inhibition of spoilage microorganisms during the ensiling process relative to the silage mixtures. These results are similar to those reported by Gülümser et al. [29], who ensiled maize mixed with soybean and cowpea. Furthermore, it was found that ensiled forage variety 1 had higher acetic and propionic acid and low butyric acid than the forage variety 2 silage. Similarly, Wilkinson and Muck [30] reported differences in organic acid concentrations of selected ensiled forage varieties when using different ensiling periods.

### 4.3. Microbial Population

Microbial community including LAB and enterobacteria plays an important role in the fermentation process during ensiling [31]. In this study, LAB production was greater in the silage mixtures than in sole forage silages, and enterobacteria counts tended to be lower in the mixed silages than in sole silages. High WSC concentrations and low pH values produced in silage mixtures likely supported high LAB populations. Di Miceli, Licata and Marceddu [32] reported a similar effect when ensiling maize planted in mixtures with forage legumes and grasses. Furthermore, ensiled forage variety 1 produced lower enterobacteria counts and higher LAB than variety 2 silages. The smaller enterobacterial populations in ensiled variety 1 forage materials could be due to well-preserved silage associated with high LAB and acetic acid and low butyric acid production. Similar results were reported by Nazar et al. [33] after exploring microbial counts of ensiled forage varieties during silage fermentation.

## 5. Conclusion

The chemical composition of the sole forage silage was lower than that of silage mixtures. Ensiled forage variety 1 produced higher nutrient composition compared to forage variety 2 silage. The results showed that ensiled forage mixtures can increase nutrient contents and give high-quality silage for ruminant feeding. The silage mixtures showed improved fermentation quality compared to sole forage silage. Forage variety 1 silage also had higher fermentation products than ensiled forage variety 2. The results revealed that ensiled forage cultivars from forage legume mixtures could improve the fermentation characteristics and make mixed crop silage a source of feed for livestock. The silage mixtures improved microbial composition than sole forage silages. In addition, microbial counts were less in ensiled forage variety 1 compared to variety 2 silage. These data indicate that the silage mixtures could give improved microbial populations and have positive effects on the silage fermentation characteristics. Additional research is required to examine the effects of silage mixtures as a source of feed for livestock in smallholder farming.

## Figures and Tables

**Table 1:** Influence of different forage systems on the chemical composition of forage maize before ensiling.

Item (% DM)	Forage systems			Maize varieties		SEM	*p* value		
	SM	MC	ML	Var 1	Var 2		FS	MV	F × M
DM	32.78^c^	35.58^b^	38.74^a^	36.05^a^	34.15^b^	0.94	0.01	0.02	0.03
CP	7.15^b^	8.58^a^	8.37^a^	7.77^a^	6.95^a^	0.86	< 0.01	0.06	0.03
NDF	34.54^a^	24.22^b^	24.88^b^	25.42^b^	28.55^a^	1.02	0.02	0.03	0.01
ADF	38.26^a^	36.62^b^	35.95^b^	38.02^b^	37.89^b^	0.89	< 0.01	0.04	0.02

*Note:* MC, maize + common vetch; var 1, Pannar 12; var 2, DKC 7372; F × M, forage system × maize varieties interaction.

Abbreviations: ADF, acid detergent fibre; CP, crude protein; DM, dry matter; FS, forage system; ML, maize + lablab; MV, maize varieties; NDF, neutral detergent fibre; SEM, standard error of mean; SM: sole maize.

^a–c^Values with different letters within the same row are significantly different (*p* < 0.05).

**Table 2:** Influence of different forage systems on the chemical composition of forage maize silages.

Item (% DM)	Forage systems			Maize varieties		SEM	*p* value		
	SM	MC	ML	Var 1	Var 2		FS	MV	F × M
DM	31.18^c^	34.65^b^	37.85^a^	34.71^a^	32.61^b^	1.08	< 0.01	0.01	0.02
CP	6.99^b^	8.15^a^	8.05^a^	7.18^a^	6.36^a^	0.76	< 0.01	0.01	0.02
NDF	33.25^a^	23.12^b^	25.72^b^	24.91^b^	27.82^a^	0.96	< 0.01	0.02	0.01
ADF	37.08^a^	35.78^b^	35.28^b^	37.21^a^	37.01^a^	1.04	0.01	0.07	0.02
WSC	11.22^c^	11.96^b^	12.71^a^	12.46^a^	11.35^b^	0.84	< 0.01	0.02	0.04

*Note*: MC, maize + common vetch; var 1, Pannar 12; var 2, DKC 7372; F × M, forage system × maize varieties interaction.

Abbreviations: ADF, acid detergent fibre; CP, crude protein; DM, dry matter; FS, forage system; ML, maize + lablab; MV, maize varieties; NDF, neutral detergent fibre; SEM, standard error of mean; SM: sole maize; WSC, water-soluble carbohydrate.

^a–c^Values with different letters within the same row are significantly different (*p* < 0.05).

**Table 3:** Influence of different forage systems on the fermentation profile of forage maize silages.

Item	Forage systems			Maize varieties		SEM	*p* value		
	SM	MC	ML	Var 1	Var 2		FS	MV	F × M
pH	3.42^a^	3.12^b^	3.08^b^	3.21^b^	3.32^a^	0.72	< 0.01	0.03	0.01
NH_3_-N (% TN)	3.73^a^	2.96^b^	3.44^ab^	3.78^b^	4.26^a^	0.92	0.01	0.02	0.03
LA (% DM)	5.94^c^	6.65^b^	7.84^a^	7.36^b^	7.18^b^	1.05	0.02	0.12	0.02
AA (% DM)	1.06^b^	1.76^a^	1.88^a^	1.81^a^	1.39^b^	0.98	< 0.01	0.01	0.03
PA (% DM)	0.84^c^	1.18^a^	1.06^ab^	1.02^a^	0.92^a^	0.88	0.01	0.15	0.01
BA (% DM)	0.46^b^	0.28^a^	0.24^a^	0.28^a^	0.35^a^	0.91	0.01	0.17	0.02

*Note*: MC, maize + common vetch; NH_3_-N, ammonia nitrogen; var 1, Pannar 12; var 2, DKC 7372; F × M, forage system × maize varieties interaction.

Abbreviations: AA, acetic acid; BA, butyric acid; FS, forage system; LA, lactic acid; ML, maize + lablab; MV, maize varieties; PA, propionic acid; SEM, standard error of mean; SM, sole maize.

^a–c^Values with different letters within the same row are significantly different (*p* < 0.05).

## Data Availability

The data used to support the findings of this study are included in the article.
